# Supplementary material for: Effect of Antibacterial Root Canal Sealer on Persistent Apical Periodontitis
Source: Antibiotics (Basel). 2021 Jun 18;10(6):741. doi: 10.3390/antibiotics10060741 (PMC8233789; doi:10.3390/antibiotics10060741)
Supplement: Supplementary file 1 [file antibiotics-10-00741-s001.zip › antibiotics-1234040-supplementary.pdf]

Effect of antibacterial root canal sealer on persistent apical periodontitis

Supplementary Materials

Synthesis of DMADDM and Modified Root Canal Sealer Preparation

In present study, DMADDM was synthesized with 2-bromoethyl methacrylate (BEMA) and 1-(dimethylamino) dodecane (DMAD). In a 20 mL scintillation vials were added 10 mmol of DMAD (Tokyo Chemical Industry), 10 mmol of BEMA, and 3 g of ethanol. A magnetic stir bar was added, and the vial was capped and stirred at 70 ◦C for 24 h. After the reaction was complete, the solvent was removed via evaporation. The number of the alkyl chain length units was 12 for DMADDM.

EndoREZ consists of root canal sealer and catalyst (optional). The main components of sealer are triethylene glycol dimethacrylate, benzoyl peroxide, diurethane dimethacrylate, GDMA phosphoric acid, bismuth oxychloride, calcium lactate pentahydrate, silicon dioxide and diacylphosphine oxide. The main components of catalyst are triethylene glycol dimethacrylate, p-toluidine diethanol, diurethane dimethacrylate, bismuth oxychloride, calcium lactate pentahydrate, silicon dioxide, deep bronze pigment and iron oxide yellow pigment. The scheme of synthesis of DMADDM and modified root canal sealer preparation is shown in Figure S1.

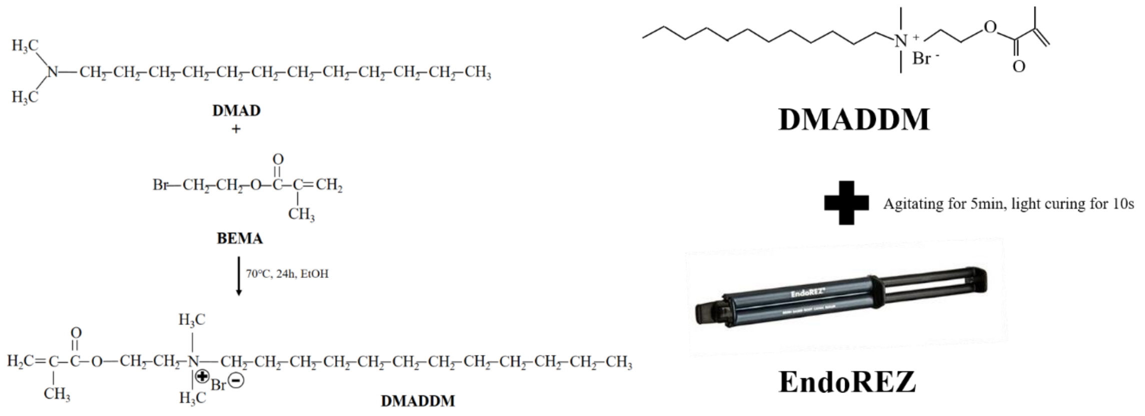

(a) (b)

Figure S1. The scheme of synthesis of DMADDM and modified root canal sealer preparation. (a) the reaction used to synthesize DMADM; (b) the scheme of modified root canal sealer preparation.

Hematoxylin and eosin stains

The maxillae and mandible of beagle dogs were fixed in 10% neutral-buffered formalin solution for 3 days, decalcified in formic acid-sodium citrate for 14 days, and embedded in paraffin. Then serial sections of 5 to 6 μm were cut from the paraffin blocks and stained with hematoxylin and eosin (H&E). Five randomly chosen visual fields were observed on each section at 400 × to calculate the number of inflammatory cells, and an ocular grid was used to quantitate cellular components at 100 intersects/field (0.06 mm2), including macrophages, lymphocytes, polymorphonuclear leukocytes (PMNs), plasma cells and neutrophils.

Calculation of the shadow volume in apical area

In this part, CT images were obtained by Morita 3D Accuitomo (Morita, Japan), and the shadow volume in apical area were measured by MIMICS V21.0 (Materialise, Switzerland). As shown in Figure S2, after transferred the CT data into MIMICS software, we identified the apical shadow in CT scan layers distributed

from top to bottom, and stained the shadow of every layer to calculate the area, then all the area data were automatically integrated to calculate the shadow volume.

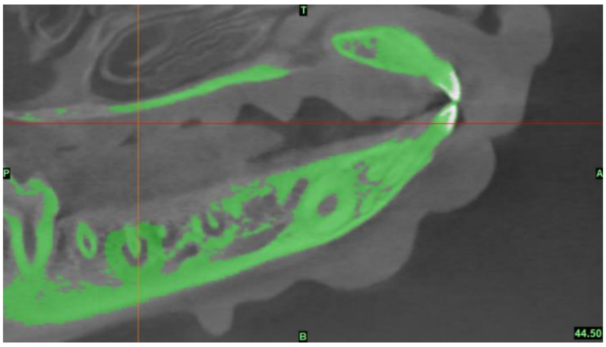

(a)

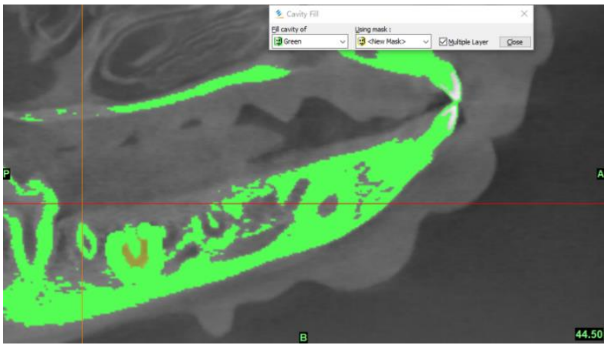

（b）

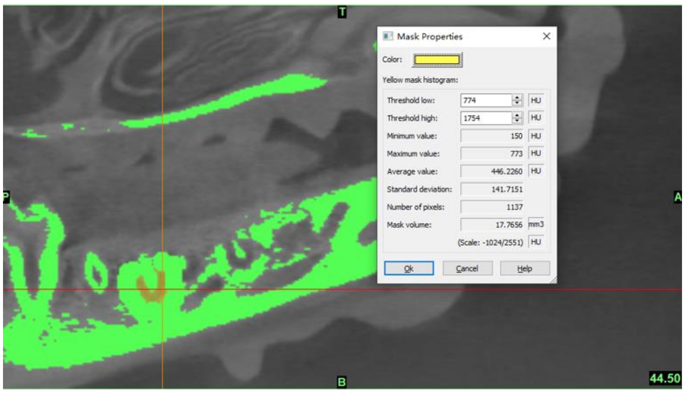

(c)

Figure S2. The measure procotol of the shadow volume in apical area. (a) one of the layer in CT images (the red arrow indicates the shadow area); (b) stained the shadow and calculate its area; (c) integrated the area of each layer to get the shadow volume.

Table S1. Histological inflammatory categories of periradicular tissue.

Categories	The amount of inflammatory cells and degree of apical bone and root destruction
0	Inflammatory cell infiltration:absent or occasional
1	Mild inflammatory infiltration with hemangiectasia and hyperemia (the average number of inflammatory cell is less than 50 in each visual fields)
2	Moderate inflammatory infiltration with apical bone and/or root resorption(the average number of inflammatory cell is 50 to 150 in each visual fields)
3	Remarkable inflammatory infiltration in the periapical area with a local abscess, as well as severe apical bone and root resorption(the average number of inflammatory cell is more than 150 in each visual fields)

Table S2. Specific primers used for real-time polymerase chain reaction.

Bacteria	Sequence (5′->3′)	Template strand
E. faecalis	F	ATTGGAAAGAGGAGTGGCGG
	R	TGAGCCGTTACCTCACCAAC
S. gordonii	F	GAGTGCTAGGTGTTAGGCCC
	R	CCTGGTAAGGTTCTTCGCGT
A. naeslundii	F	CTCGACACCGTGAAGTTGGA
	R	CGACTTCGTCCCAATCACCA
L. acidophilus	F	TGGGGAACCTGCCCCATAG
	R	GGTAGGCCGTTACCCTACCA
